# A Dietary Supplement in the Management of Patients with Lumbar Osteochondrosis: A Randomized, Double-Blinded, Placebo-Controlled Study

**DOI:** 10.3390/nu16162695

**Published:** 2024-08-14

**Authors:** Brenda Laky, Daniel Huemer, Martin Eigenschink, Benedikt Sagl, Rainer Thell, Karl-Heinz Wagner, Werner Anderl, Philipp R. Heuberer

**Affiliations:** 1 Austrian Research Group for Regenerative and Orthopedic Medicine (AURROM), 1050 Vienna, Austria; danielhuemer.medical@gmail.com (D.H.); martin.eigenschink@gmail.com (M.E.); werner@anderl.at (W.A.); philipp@heuberer.at (P.R.H.); 2 Austrian Society of Regenerative Medicine (RegMed), 1010 Vienna, Austria; 3 Faculty of Medicine, Sigmund Freud Private University Medicine, 1020 Vienna, Austria; 4 Center for Clinical Research, University Clinic of Dentistry, Medical University of Vienna, 1090 Vienna, Austria; 5 Department of Nutritional Sciences, University of Vienna, 1090 Vienna, Austria; karl-heinz.wagner@univie.ac.at; 6 Medical University of Vienna, 1090 Vienna, Austria; rainer.thell@gesundheitsverbund.at; 7 Department for Trauma and Orthopedic Surgery, AUVA Trauma Center Vienna-Meidling, 1100 Vienna, Austria; 8 Competence Center Artificial Intelligence, University Clinic of Dentistry, Medical University of Vienna, 1090 Vienna, Austria; benedikt.sagl@meduniwien.ac.at; 9 Emergency Department, Klinik Donaustadt, 1220 Vienna, Austria; 10 Momentum Praxis Mödling, 2340 Mödling, Austria; 11 OrthoCare, 1100 Vienna, Austria; 12 HealthPi Medical Center, 1010 Vienna, Austria

**Keywords:** lower back pain, magnetic resonance images (MRI), collagen type II, hyaluronic acid, n-acetyl-glucosamine, bamboo extract, L-lysine

## Abstract

Various nutritional supplements are available over the counter, yet few have been investigated in randomized controlled trials. The rationale for using the specific mix of nutritional substances including collagen type II, hyaluronic acid, n-acetyl-glucosamine, bamboo extract, L-lysine, and vitamin C is the assumption that combining naturally occurring ingredients of the intervertebral disc would maintain spine function. This double-blinded, placebo-controlled randomized trial aimed to evaluate the efficacy of a nutraceutical supplement mix in the management of lumbar osteochondrosis. Fifty patients were randomly assigned to either the supplement or placebo group in a 1:1 ratio. Patient-Reported Outcome Measures (PROMs) included the Oswestry Disability Index (ODI), the visual analogue scale for pain (pVAS), short form-12 (SF-12) physical and mental component summary subscale scores (PCS and MCS, respectively), and global physical activity questionnaire (GPAQ). Magnetic resonance imaging (MRI) was used to evaluate degenerative changes of intervertebral discs (IVD) including Pfirrmann grades as well as three-dimensional (3D) volume measurements. Data were collected at baseline and after the 3-month intervention. None of the PROMs were significantly different between the supplement and placebo groups. Disc degeneration according to Pfirrmann classifications remained stable during the 3-month intervention in both groups. Despite no significance regarding the distribution of Pfirrmann grade changes (improvement, no change, worsening; *p* = 0.259), in the supplement group, one patient achieved a three-grade improvement, and worsening of Pfirrmann grades were only detected in the placebo group (9.1%). Furthermore, in-depth evaluations of MRIs showed significantly higher 3D-measured volume changes (increase) in the supplement (+740.3 ± 796.1 mm^3^) compared to lower 3D-measured volume changes (decrease) in the placebo group (−417.2 ± 875.0 mm^3^; *p* < 0.001). In conclusion, this multi-nutrient supplement might not only stabilize the progression of lumbar osteochondrosis, but it might also potentially even increase IVD volumes as detected on MRIs.

## 1. Introduction

Lumbar osteochondrosis, also referred to as osteochondritis dissecans, is generally related to degeneration of the intervertebral disc (IVD) and is a major contributor to lower back pain, loss of function, and work absenteeism in the industrialized world. The primary etiology of lumbar osteochondrosis remains poorly understood, but multifactorial causes including hereditary, anatomical, traumatic, nutritional, inflammatory, and metabolic factors are likely to be contributors to the progression of such a joint disorder [1]. Current non-surgical therapies for lumbar osteochondrosis to decrease pain and return patients to normal function include pharmaceutical pain therapy (e.g., analgesics, non-steroidal anti-inflammatory drugs, muscle relaxants, opiates), physical therapy (e.g., physiotherapy, electrotherapy, ultrasound, heat treatment), and treatment with injections (e.g., trigger point injections, nerve blocks, hydrogel). While an appropriate diet alone might not hinder lumbar osteochondrosis development, specific nutritional management might prolong the progression of such a joint disease.

**Figure 1:**
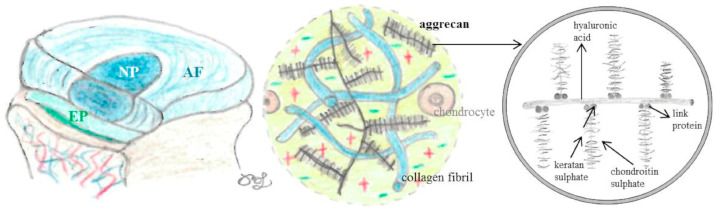
Schematic images of intervertebral discs (IVD) including structures and compositions adopted from [2]. The nucleus pulposus (NP), the annulus fibrosus (AF), and the endplate (EP) consist of collagen and proteoglycans. Aggrecan, a large proteoglycan, is composed of hyaluronic acid, chondroitin, and keratan sulfate.

IVDs are composed of the nucleus pulposus (NP), the annulus fibrosus (AF), and the endplate (EP). The macro- and micro-structure of IVDs are presented in Figure 1.

**Figure 2:**
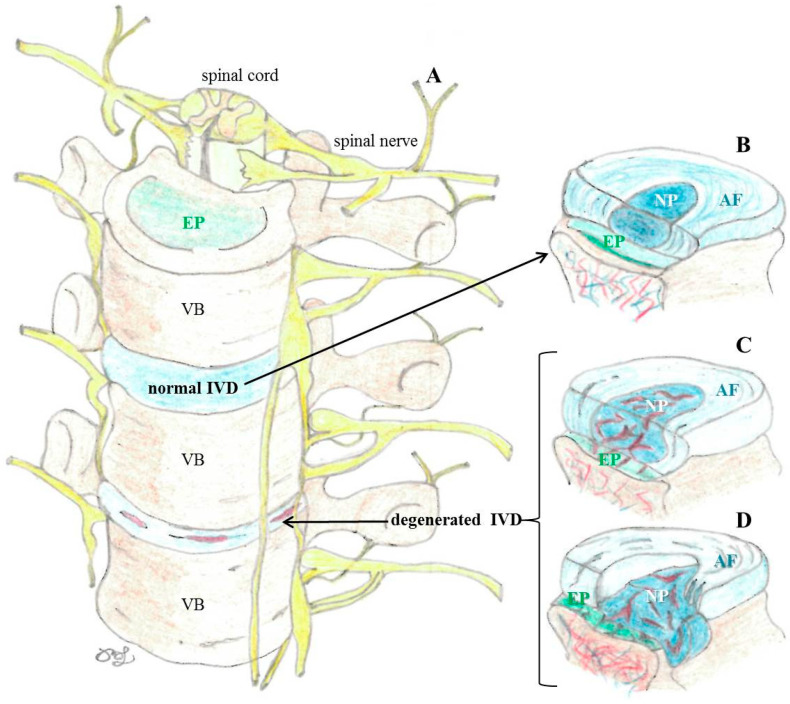
Schematic images of spinal column (**A**) adopted from [2]; normal intervertebral discs (IVD; (**B**)), degenerated IVD (**C**), and herniated IVD (**D**).

The NP, the shock-absorbing gelatinous and highly hydrated center of the IVD, mainly consists of a disorganized conglomeration of hydrophilic proteoglycans (mainly aggrecan) interspersed with type II collagen fibers. The NP is surrounded by the complex fibrous (consists of highly organized type I collagen layers) and fibrocartilaginous (contains chondrocytes) lamellar structure of the AF. The outermost layer of the AF is the only site, where the IVD is slightly vascularized, while the inner layers of the AF as well as the NP are without innervation and vascular supply, therefore nutrients are only received via diffusion. The vascularized and cartilaginous EP consists of fibrocartilage towards the AF and hyaline cartilage towards the vertebral body. The structure and physiology of IVDs are altered in degeneration (Figure 2).

The rationale for using a specific mix of nutritional supplements including a collagen hydrolysate including collagen type II, mucopolysaccharides, hyaluronic acid, n-acetyl-glucosamine, bamboo extract, L-lysine, and vitamin C was the assumption that combining naturally occurring ingredients of the IVD including proteins (e.g., collagen) and proteoglycans (e.g., aggrecan) would maintain spine function regarding the structure and possible inflammatory balancing aspects.

Collagen, a fibrous structural protein of the connective tissue, strengthens the tendons and skeletal muscles as well as the ligaments supporting the vertebral column. The amino acids proline (non-essential) and lysine (essential) as well as ascorbic acid (vitamin C) are involved in collagen synthesis. Next to vitamin C’s essential role in, e.g., proline and lysine hydroxylase, is its anti-oxidative capacity. A recent study including data from more than 4700 individuals reported associations between vitamin C and spinal pain [3]. Aggrecan is a cartilage-specific proteoglycan in the cartilage matrix and synovial fluid, which consists of the glycosaminoglycans (GAG) hyaluronic acid, and keratan- and chondroitin-sulfate, a core and a link protein, respectively. GAGs are essential for absorbing and storing large volumes of water in the NP of the IVD, and thus function as shock absorbers.

Numerous studies have demonstrated that oral administration of collagen hydrolysate [4,5] and the GAGs hyaluronic acid [6] and glucosamine [7] are able to reduce articular pain in the knee. Controversies over the efficacy of orally administered dietary compounds could be ruled out by preclinical and clinical studies, which demonstrated that the hydrolyzed form of collagen is able to cross—without enzymatic cleavage and, therefore, as a complete peptide—the intestinal barrier and enter the blood circulation system, further accumulate in cartilage tissue, and stimulate production of type II collagen and proteoglycans in the extracellular matrix of cartilage [8,9,10]; orally administered hyaluronic acid also reaches the joint [11]; and glucosamine supplementation to healthy volunteers and patients with osteoarthritis increases its concentrations in the synovial fluid and plasma [12,13].

Supplementation of such basic IVD components may be beneficial, especially if balances between anabolic and catabolic processes are disturbed. Next to their ability as essential elements of the cartilage to stimulate anabolic processes, inflammation- and oxidative stress-related catabolic processes in the cartilage may also be modulated by such nutraceuticals [14]. Although most of the research was conducted in the knee joint, it is also known that IVD height narrowing is increased in patients with knee osteoarthritis [15].

Bamboo extract is a source of (a) natural antioxidants (polyphenols and flavonoids, e.g., chlorogenic acid, caffeic acid, ferulic acid, p-coumaric acid, orientin, homoorientin, vitexin, and isovitexin), which may prevent oxidative damage caused by reactive oxygen species (ROS), such as superoxide, hydroxyl, and peroxyl radicals; and (b) natural silicic acid (at least 75%), which may contribute to the elasticity of the connective tissue as a cross-linking between mucopolysaccharides (collagen and elastin) to ensure stability. In an in vitro investigation, bamboo extract inhibited lipotoxicity-induced interleukin 6 (IL-6, an inflammatory cytokine) overproduction in muscle and adipose cell lines [16]. Yet, in vivo studies providing evidence regarding their potential antioxidant capacity and ability to ensure cartilage stability are missing.

Despite conflicting and weak evidence and reasonable scientific rationale regarding efficacy, nutritional supplements are frequently used and anecdotally offered in clinical practice. Notification, registration, or declaration of dietary supplements has not been required since the commencement of the act in Austria according to the Austrian Agency for Health and Food Safety. Even though dietary supplements do not fall under the drug or medical products law, the trial was conducted accordingly.

Given the plausible rationale that dietary interventions are of low risk, studies investigating such non-surgical treatment options are rare. To our knowledge, there are no published clinical trials that evaluated the effectiveness of such a specific nutritional supplement when compared to a placebo and carried out in a proper randomized trial set-up.

The overall goal of this randomized placebo-controlled, double-blinded trial (RCT) was to evaluate the efficacy of a nutraceutical supplement, which is a combination of specific dietary compounds including collagen type II, hyaluronic acid, n-acetyl-glucosamine, bamboo extract, L-lysine, and vitamin C in the management of lumbar osteochondrosis. The primary objective was to determine if a 3-month supplementation can improve pain and function according to the Oswestry Disability Index (ODI) in the management of patients with lumbar osteochondrosis. Secondary objectives included comparisons between intervention and placebo regarding various measures of intervertebral disc distances according to magnetic resonance images (MRI); visual analogue scale for back pain (VAS), quality of life questions using the short form-12 (SF-12) physical and mental component summary subscale scores (PCS and MCS, respectively), global physical activity questionnaire (GPAQ), and a global assessment regarding efficacy/satisfaction with the interventional procedure.

## 2. Materials and Methods

### 2.1. Study Design

This was a double-blinded, randomized, placebo-controlled study carried out according to the principles of the Declaration of Helsinki, Good Clinical Practice (GCP), as well as all national legal and regulatory requirements. It followed the Consolidated Standards of Reporting Trials (CONSORT statement) [17] (Supplementary Table S1). The study was first approved by the ethics committee of the Medical University of Vienna (Austria) on 10 January 2017 (EC Nr: 1998/2016; protocol version 1.0). The ethics committee additionally approved two protocol amendments until 12 March 2019 (EC Nr: 1998/2016; protocol version 1.4). The study was registered at the ISRCTN (ISRCTN17230715).

### 2.2. Participants

All patients presenting with symptomatic MRI-confirmed lumbar osteochondrosis at the private practice were screened for eligibility by orthopedic consultants (P.H., W.A.). If meeting the inclusion criteria (Supplementary Tables S2 and S3 list all inclusion and exclusion criteria for participation), they were informed and invited to participate in the trial. A recruitment log for all potential patients including those who were ineligible according to in- and exclusion criteria, who declined to enter the study, and those who refused/rejected participation after consenting to be randomized was recorded.

Once the patient signed the informed consent, the participant was randomized to one of the two arms. Randomization was performed in a 1:1 ratio and generated by GraphPad statistical software (online version 2016, QuickCalcs, La Jolla, CA, USA). All packages were labeled with the randomization number. Participants and treating orthopedic surgeons were blinded to group allocation during the study.

### 2.3. Intervention

The active study multi-nutrient supplement capsules (Vertebene Bandscheibenkapseln, Natural Products & Drugs GmbH, Spittal an der Drau, Austria) are composed of a mix of the following nutritional compounds:

- collagen hydrolysate (300 mg/capsule);
  ○ collagen type II (180 mg/capsule);
  ○ mucopolysaccharides (90 mg/capsule);
  ○ hyaluronic acid (30 mg/capsule);
- n-acetyl-glucosamine (100 mg/capsule);
- bamboo extract (70 mg/capsule);
- L-lysine hydrochloride (25 mg/capsule);
- vitamin C (30 mg/capsule);
- other ingredients: gelatin, magnesium stearate.

The same company (Natural Products & Drugs GmbH, Austria) producing the active capsules provided the control capsules (placebos). Cellulose was used as an inactive filler for the placebo capsules. The active and placebo capsules were encapsulated in hydroxypropylmethylcellulose capsules, were similar in appearance, taste, and smell, and were provided in identical sealed containers with identical labeling providing study information and the coded randomization number.

Patients in both groups were advised to take 2 × 2 capsules daily with a glass of water (2 capsules during breakfast and 2 capsules during dinner) for three months. The rationale for the 3-month supplementation was based on knee studies reporting pain reduction after 12 weeks of glucosamine administration [18,19,20]. Additionally, patients were provided with an intervention manual, which included suggestions for physical exercises. Concomitant medication was allowed as long as the dose remained stable from screening to the end of the study. During the entire study period, subjects were not allowed to consume any other nutritional supplements similar to the study product. All patients in both groups were told to avoid drastic changes in their diet and lifestyle; otherwise, usual care for their symptoms was provided.

### 2.4. Outcomes

Patient demographics including age, gender, weight, height, dietary, and symptom-related information were collected from the participants through an anamnestic questionnaire. The body mass index (BMI) was calculated as weight (kg) divided by height (m^2^) and was classified as underweight (BMI < 18.5 kg/m^2^), normal weight (18.5 ≤ BMI < 25.0 kg/m^2^), overweight (25.1 ≤ BMI < 29.9 kg/m^2^), and obese class I (30.0 ≤ BMI < 34.9 kg/m^2^), class II (35.0 ≤ BMI < 39.9 kg/m^2^), class III (BMI ≥ 40.0 kg/m^2^).

#### 2.4.1. Patient-Reported Outcome Measures (PROMs)

Patient-Reported Outcome Measures (PROMs) included the German version 2.1 of the Oswestry Disability Index (ODI) [21,22] to assess functional limitations specifically for patients with disorders of the spine. The ODI is a 10-item questionnaire with ordered response items ranging from 0 to 5 points for each question. The questions address physical function, sleep, home/work function, and social life. Points of each question were summed up (one missing item was allowed) and the percentage of the maximum possible score (45–50 points) was calculated (final range 0–100%). Higher scores indicate greater dysfunction; the visual analogue scale for back pain (VAS) ranging from no (0) to severest pain (100) on a 10 cm scale; the quality-of-life score Short Form 12 (SF-12) [23] incorporating a physical and mental component summary subscale scores (PCS and MCS, respectively) with 0 to 100 points each and higher scores indicating better health status; the Global Physical Activity Questionnaire (GPAQ) [24] assesses occupational, active commuting, and recreational physical activity as well as during siting. The intensity of physical activity was expressed as metabolic equivalents (METs) and total physical activity in MET-minutes per week was computed for each setting; a global assessment of satisfaction with the interventional procedure was measured on a Likert scale with five categories ranging from very satisfied (4 points) to not satisfied at all (0 points).

#### 2.4.2. Radiological Assessment

All MRIs taken before intervention were routinely evaluated by a radiologist and orthopedic surgeon for diagnosis. A study-specific analysis of all lumbar spine MRIs was conducted and included an evaluation according to Pfirrmann et al. [25] classification system considering disc signal intensity, structure, and height, as well as a distinction between NP and AF (Supplementary Table S4). Each lumbar IVD (from L1-L2 to L5-S1) was assigned to a single Pfirrmann grade. Pfirrmann grades ≥III were defined as disc degeneration [26]. To calculate and quantify volume changes, three-dimensional (3D)-based volume calculations were carried out using the radiological image processing software ITK-Simpleware Navigation Analysis and Display (SNAP; version 3.8.0). ITK-SNAP is free software in DICOM format with a primary focus on 3D visualization of anatomical structures in medical imaging. In each slice of the MRI, the region of interest (ROI) was manually outlined in the sagittal plane. By multiplying all 2D areas with the specific slice thickness of the MRI, a 3D model of the IVD was created and the volume (in mm^3^) for each IVD was calculated.

All MRI assessors were blinded to any patient-related data. Despite performing inter- and intra-observer agreement analyses, in cases of grading discrepancies, a consensus agreed to by all raters was obtained for final data analysis.

All in-depth radiological assessments were part of the thesis publication [27] from a co-author (D.H.).

#### 2.4.3. Adverse Events

Interim analysis or early discontinuation of the study was not considered necessary since patients were not exposed to any potentially harmful therapies. No additional risks were expected with dietary supplements. However, any unfavorable change from patients’ preinterventional status (e.g., concomitant illnesses) regardless of any possible relationship to intervention was recorded as an adverse event. If considered necessary, the treating orthopedic consultant was able to exclude participants for the health of the patient.

### 2.5. Statistical Analysis

#### 2.5.1. Sample Size

The a priori sample size calculation was based on previously reported data of the primary endpoint (ODI [21,22]). Calculations determined that at least 45 patients per group would achieve 80% power to detect an 8-point reduction in ODI with an estimated standard deviation of 15 for a 2-sided test with a significance level α of 0.05 between the dietary supplement and placebo. To compensate for an expected loss of recruited patients during follow-up, the number of participants planned for enrolment before the study started was a total of 100 patients with 50 patients in each group. Additionally, to the primary endpoint, we performed an a priori sample size calculation for the most important secondary endpoint using previously reported data of IVD distances [28]. Calculations determined that at least 16 patients per group would achieve 90% power to detect a 3 mm change of IVD distances with an estimated standard deviation of 2.5 mm for a 2-sided test with a significance level α of 0.05 between the supplement and placebo group.

Unfortunately, due to recruitment problems caused by various relocations of the investigators (B.L., P.H., W.A.) as well as the COVID-19 pandemic situation, we were not able to continue enrollment to reach 50 patients per group. However, post-hoc power analysis revealed that the study was powered at 99.5% (effect size = 1.38) to detect significant 3D-measured volume changes from before to after the 3-month intervention between the supplement and placebo group.

#### 2.5.2. Statistical Analysis

Summary statistics described the demographic and clinical characteristics of participants. The distribution of continuous data was assessed by a visual inspection of histograms and the Kolmogorov–Smirnov test. Normally distributed quantitative data were described by means and standard deviations (SD), otherwise as medians and ranges. Categorical data were described using frequency counts and percentages.

For normally distributed continuous variables, the independent *t*-test and the Mann–Whitney U-test for non-parametric variables were performed to compare intervention groups. Paired *t*-tests and Wilcoxon signed-rank tests were used to compare parametric and nonparametric variables, respectively between pre- and postoperative assessments. Chi-square tests were employed to examine the relationship between proportions and frequency counts of categorical variables and treatment groups.

Cohen’s Kappa statistics were used to evaluate inter-rater reliability (between D.H. and P.H.) for all Pfirrmann grades. Inter-rater reliability is presented as Cohen’s Kappa (κ) and its 95% confidence intervals (95% CI). Intra- (two measurements at different time points one month apart by one rater, D.H.) and inter-rater (two raters; D.H. and P.H.) reliability of MRI-volumes were evaluated for a subset of 10 patients with intraclass correlation coefficient (ICC) and its 95% CI. Cohen’s Kappa and ICC values were classified as poor (< 0.20), weak (0.21–0.40), moderate (0.41–0.60), strong (0.61–0.80), and very strong consistency (0.81–1.0).

Statistical significance was reported at a *p*-value of <0.05 level (two-sided). All data were analyzed using SPSS software (PAWS Statistics 26; SPSS Inc., Chicago, IL, USA).

## 3. Results

**Figure 3:**
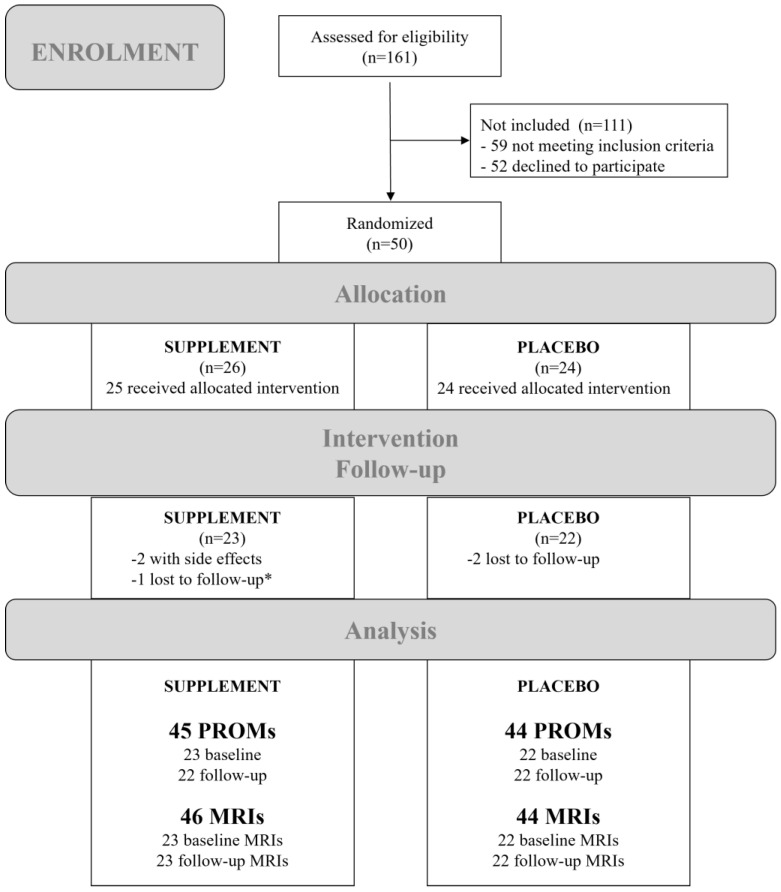
Participant flowchart showing the numbers of participants who were recruited, randomly assigned, dropped out, and analyzed during the trial. * The patient, who was wrongly assigned to the supplement group, dropped out for follow-up. Abbreviations: MRIs, magnetic resonance images; PROMs, patient-reported outcome measures.

A total of 161 participants were recruited and assessed for eligibility between 3 January 2017 and 9 January 2021 of which 59 did not meet the inclusion criteria and 52 declined to participate. Hence, leaving 50 participants who were randomly assigned to receive either the supplement (n = 26) or placebo (n = 24). One patient was wrongly assigned to the supplement group; however, this patient dropped out for follow-up. Two further participants were uncontactable for follow-up or withdrew consent. Two patients from the supplement group were excluded during the intervention due to minor side effects. In both cases, women reported some kind of nausea, which was recovered by stopping the supplement. The study was completed by the remaining 45 participants (23 in the supplement and 22 in the placebo group). The participant flowchart is presented in Figure 3.

### 3.1. Baseline Characteristics

There were no significant differences in patients’ baseline characteristics between the two study groups, except regarding reported allergies (pollen/dust/animal hair: 3 vs. 5; drugs: 1 vs. 6; chemicals: 1 vs. 2 times in the supplement and placebo group, respectively), which was significantly more often reported in the placebo group (Table 1).

### 3.2. Patient-Reported Outcome Measures (PROMs)

Significant differences between the supplement and placebo groups were only detected regarding baseline physical activity. Interestingly, significant placebo-VAS for back pain improvements were reported by patients. No other significant differences were found regarding PROMs (Table 2).

### 3.3. Radiological Assessment

A total of 450 IVDs from pre- and post-intervention MRIs were assessed. There was excellent agreement in terms of MRI IVD-volumes (intra-rater ICC = 0.993 (95% CI: 0.988–0.996), *p* < 0.001 and inter-rater ICC = 0.995 (95% CI: 0.980–0.998), *p* < 0.001) and all Pfirrmann classifications showed strong or very strong agreements (Cohen’s Kappa was between 0.749 and 0.944; *p* < 0.001; see Supplementary Table S4).

#### 3.3.1. Pfirrmann Grade of Disc Degeneration

Disc degeneration remained stable from before to after the 3-month intervention in both groups (Table 3). No significant differences regarding disc degeneration were observed between the supplement and placebo group at both time points. Distributions of Pfirrmann grades of each IVD are presented in Supplementary Table S5.

Despite no significance regarding the distribution of Pfirrmann grade changes (improvement, no change, worsening; *p* = 0.259), in the supplement group, one patient achieved a three-grade improvement and on the other hand, worsening of Pfirrmann grades was only detected in the placebo group (Table 4).

#### 3.3.2. 3D-MRI Measures

Comparisons between the supplement and placebo groups showed no significant differences regarding the volumes of each IVD distance (Supplement Table S6) nor regarding the means of the IVD distances (Table 5).

**Figure 4:**
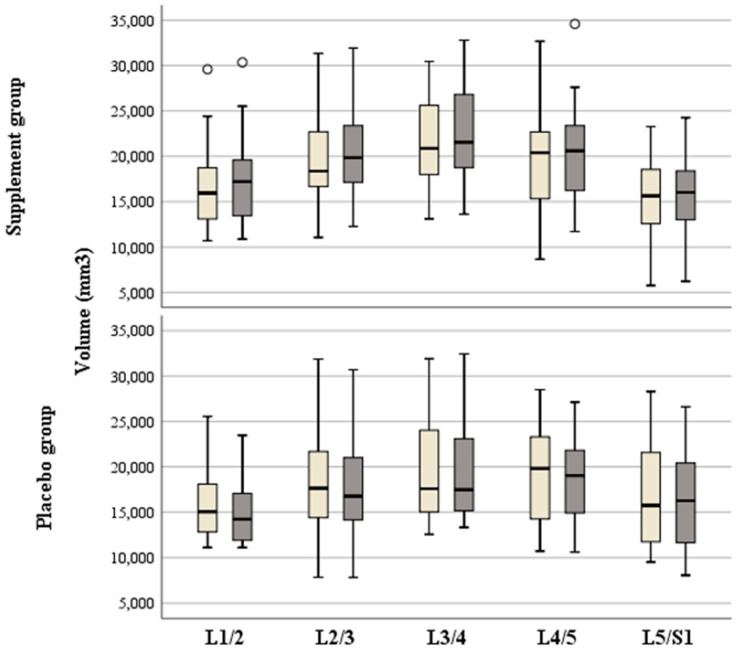
Box plots showing volumes of the intervertebral disc distances before (light gray) and after (dark gray) the 3-month intervention of the supplement and placebo groups. Circles indicate outliers.

However, while all volumes in the supplement group increased, all volumes in the placebo group decreased from before to after the intervention (Supplement Table S6). The improvement in the supplement group was significantly better regarding L1-L2 (*p* < 0.001), L2-L3 (*p* = 0.005), L3-L4 (*p* < 0.001), L4-L5 (*p* = 0.019), but not regarding L5-S1 (*p* = 0.062). All volumes of each IVD distance and comparisons between and within groups are presented in Figure 4 and Supplementary Table S6.

**Figure 5:**
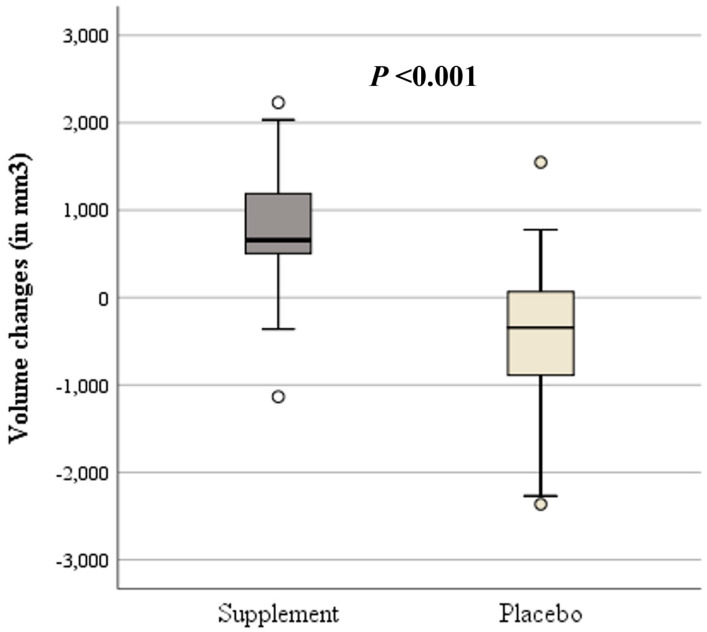
Box plots showing volume changes from before to after the 3-month intervention of the supplement (dark gray) and placebo group (light gray). Circles indicate outliers.

Indeed, a comparison between volume changes from before to after the 3-month intervention showed significantly higher volume changes (increase) in the supplement (+740.3 ± 796.1 mm^3^) compared to lower volume changes (decrease) in the placebo group (−417.2 ± 875.0 mm^3^; *p* < 0.001; Figure 5).

## 4. Discussion

This paper presents the first randomized, double-blinded, placebo-controlled study investigating the potential effect of a specific nutraceutical on lumbar osteochondrosis. And as such it marks an important step for a better understanding of the clinical relevance of supplements. Although this RCT did not find significant differences between the supplement and placebo group regarding PROMs, disc degeneration according to Pfirrmann classifications remained stable during the 3-month intervention in both groups. Furthermore, in-depth evaluations of MRIs showed significantly higher 3D-measured volume changes (increase) in the supplement (+740.3 ± 796.1 mm^3^) compared to lower 3D-measured volume changes (decrease) in the placebo group (−417.2 ± 875.0 mm^3^; *p* < 0.001).

According to the clinical practice guideline from the American College of Physicians (ACP) developed an evidence-based guideline [29] with clinical recommendations on non-invasive treatment for low back pain, there was no mention in the systemic review of evidence regarding any dietary supplement as a non-invasive treatment option. This is not surprising as studies evaluating nutrition-related supplements for chronic back pain are sparse [30,31,32,33,34].

The efficacy of vitamin D supplements is often reported but is still controversial. While a meta-analysis [32] reported a vitamin D deficiency to be associated with low back pain, a more recent large UK cohort study showed no association between vitamin D status (21.6% prevalence of vitamin D deficiency) nor with vitamin D supplements (only 4.0% took vitamin D supplements) and low back pain [34].

However, comparing ODI between our findings and interventions with or without vitamin D3 supplementation of patients with non-surgically treated symptomatic lumbar spinal stenosis [35], there was similar pre-intervention ODI. Post-intervention ODIs were significantly lower in the supplement (n = 26; 13.8 ± 6.6) than in the non-supplement group (n = 25; 17.8 ± 6.9; *p* = 0.038), which was not comparable to our findings. Hence, it seems that vitamin D3 supplementation is more successful than our multi-nutrient supplement without vitamin D3 regarding spine function. Pain was in general higher at baseline (almost 70% in both groups) in this vitamin D3 intervention study compared to our findings (around 40% in both groups). However, vitamin D3-group post-intervention pain levels were around 30% and thus, similar to our findings (in both groups), but were significantly different to their controls (*p* = 0.027). According to the ACP guidelines mean between-group differences regarding ODI as well as VAS, small effects were defined as 5–10 points and moderate as 11–20 points [29]. Applied to our RCT, we only reached a small effect regarding VAS improvements from baseline (approximately 7 points), but the mean between-group differences were neglectable. The study by Ko et al. [35] showed that despite being significantly different, there was also no effect regarding the ODI (4 points), while on the other a moderate effect was detected regarding VAS (between-group difference was 13.7 points). However, we are unaware of the vitamin D status in our cohort, and thus, a comparison to a vitamin D-deficient cohort may be biased. Furthermore, treatment regimens especially regarding vitamin D supplementation need to be considered.

Literature shows that various conservative therapeutic approaches are effective in reducing both, functional limitation and pain intensity for lumbar osteochondrosis. Physiotherapy [36] and acupuncture [37] show the greatest improvements in terms of both ODI and VAS. Manual therapies [38] and drug treatments [39] showed only moderate improvements, with drug therapies primarily providing short-term pain relief. Preventive measures such as back and ergonomic training [40,41] are also valuable, especially for long-term health. The combined results of the ODI and the VAS provide a comprehensive picture of the therapeutic effects: While the ODI documents the effects on quality of life and functional abilities, the VAS captures the immediate intensity of pain. Future studies should use both instruments to ensure a holistic assessment of the therapeutic effects and to investigate the optimal combination and individualization of conservative treatment approaches.

Furthermore, it is worth mentioning that the placebo group was significantly more physically active at baseline, despite being slightly older than the supplement group. However, it also showed that the supplement group was able to increase physical activity, while physical activity decreased in the placebo group.

Unfortunately, we are unable to explain why the placebo compared to the supplement group reported a significant reduction in VAS pain. However, the analgesic placebo effect for various diseases is well documented [42].

Compared to findings regarding PROMs and Pfirrmann gradings, which are known as the gold standard to evaluate MRIs, significant improvements could be detected with the 3D measurement method (volume). Indeed, it has been reported that Pfirrmann grading lacks sensitivity to change and that the scale has only moderate to good reliability [43,44]. However, according to our knowledge, there is no study reporting a Pfirrmann 3-grade improvement, and publications regarding 3D measurements are rare in the clinical setting.

The main limitation of this study is that the sample size is small, and the duration of the intervention might be too short to be able to detect significant differences regarding PROMs as self-reported questionnaires are less sensitive than in-depth MRI measurements. Another limitation is that only routinely obtained MRIs were available for the study; these MRIs (from low tesla MRI scanners instead of e.g., seven tesla MRI scanners) were not optimal for the 3D measurement in terms of slice thickness and resolution. In the future, more complex MRI sequences should be used for measurements. Furthermore, no blood testing was performed, which could be regarded as a more objective tool regarding the bioavailability of nutrients in such a multi-nutrient supplement. Hence, future studies would definitely benefit from a larger sample size to further explore the multi-nutrient supplement’s efficacy especially when investigating longer interventions (e.g., 6 or even 12 months).

However, the primary strength of our study is the study design of a randomized, double-blinded, placebo-controlled trial to investigate the effects of a multi-nutrient supplement in the management of patients with lumbar osteochondrosis.

## 5. Conclusions

In summary, this multi-nutrient supplement might not only stabilize the progression of lumbar osteochondrosis, but it might also potentially increase IVD volumes as detected on MRIs. However, our results must be considered preliminary until such effects are proven by randomized controlled trials with larger sample sizes and longer supplement interventions.

## Figures and Tables

**Table 1:** Baseline characteristics of participants.

Variable	Supplement (n = 23)	Placebo (n = 22)	*p* Value
Age (years)	52.7 ± 10.4	56.5 ± 14.5	0.331 ^1^
Gender (n, %) Female Male	10 (43.5) 13 (56.5)	14 (63.6) 8 (36.4)	0.236 ^2^
BMI (kg/m^2^)	26.8 ± 4.4	27.4 ± 3.9	0.645 ^1^
normal weight (18.5–25.0) overweight (25.1–29.9) obese (30.0–34.9)	8 (34.8) 8 (34.8) 7 (30.4)	8 (36.4) 7 (31.8) 7 (31.8)	0.978 ^2^
Smoking status (n, %) non-smoker ex-smoker current smoker	7 (31.8) 13 (59.1) 2 (9.1)	8 (34.8) 12 (52.2) 3 (13.0)	0.867 ^2^
Diet (n, %) omnivore only or mainly vegetarian	20 (87.0) 3 (13.0)	20 (90.9) 2 (9.1)	0.673 ^2^
Allergy (yes, %)	5 (21.7)	13 (59.1)	0.016 ^2^
Disease (yes, %)	12 (52.2)	15 (75.0)	0.206 ^2^
Medication (yes, %)	13 (56.5)	13 (59.1)	0.862 ^2^

Abbreviations: BMI, body mass index. Values of continuous variables are presented as means ± standard deviation. *p* values were obtained using ^1^ student *t*-test and ^2^ chi-squared tests.

**Table 2:** Patient-Reported Outcome Measures (PROMs).

Variable	Supplement (n = 23 *)	Placebo (n = 22)	*p* Value (between Groups)
Oswestry Disability Index (ODI, %)			
before intervention after intervention	22.2 (6.0–48.0) 17.0 (4.0–42.2)	18.0 (4.0–60.0) 16.0 (2.0–46.0)	0.750 ^1^ 0.860 ^1^
*p*-value (within groups)	0.092 ^2^	0.121 ^2^	-
ODI improvement	2.1 (−8.0 to 16.9)	2.0 (−15.9 to 28.9)	0.906 ^1^
Visual analogue scale for back pain (VAS, %)			
before intervention after intervention	40 (10–80) 35 (3–70)	40 (5–80) 30 (0–70)	0.782 ^1^ 0.812 ^1^
*p*-value (within groups)	0.129 ^2^	0.019 ^2^	-
VAS improvement	−7.5 (−50.0 to 25.0)	−6.0 (−40.0 to 20.0)	0.461 ^1^
Quality-of-life score Short Form 12 (SF-12)			
PCS before intervention PCS after intervention	42.8 (24.4–53.1) 43.5 (19.7–51.7)	43.1 (22.1–57.2) 44.0 (23.0–53.5)	0.847 ^1^ 0.542 ^1^
*p*-value (within groups)	0.615 ^2^	0.592 ^2^	-
PCS improvement	0.34 (−13.2 to 14.2)	−0.26 (−23.4 to 9.4)	0.542 ^1^
MCS before intervention MCS after intervention	52.1 (22.5–65.3) 55.9 (29.6–62.2)	51.6 (29.8–64.0) 54.6 (36.6–68.7)	0.847 ^1^ 0.869 ^1^
*p*-value (within groups)	0.236 ^2^	0.053 ^2^	-
MCS improvement	−1.3 (−11.9 to 9.9)	−1.9 (−19.1 to 8.0)	0.796 ^1^
Global Physical Activity Questionnaire (GPAQ; MET-min/week)			
before intervention after intervention	1940 (0–27,360) 2130 (0–11,640)	5920 (240–30,000) 4800 (0–28,200)	0.036 ^1^ 0.056 ^1^
*p*-value (within groups)	0.341 ^2^	0.341 ^2^	-
PA improvement	−100 (−24,120 to 11,040)	−960 (−5760 to 6680)	0.778 ^1^

Abbreviations: MCS, mental component summary; MET, metabolic equivalent task (MET); PCS, physical component summary. * One follow-up questionnaire was not available. Values are presented as medians and ranges. *p* values were obtained using ^1^ Mann–Whitney U test and ^2^ Wilcoxon signed-rank tests.

**Table 3:** Disc degeneration including Pfirrmann grade III, IV, and V.

Variable	Supplement (23 Patients 115 IVDs)	Placebo (22 Patients 110 IVDs)	*p* Value (between Groups)
Disc degeneration * (n, %)			
before intervention after intervention	61 (53.0) 53 (46.1)	59 (53.6) 52 (47.3)	0.929 ^1^ 0.859 ^1^
*p*-value (within groups)	0.291 ^1^	0.345 ^1^	-

Abbreviations: IVDs, intervertebral discs. * Pfirrmann grade III + IV + V. Values are presented as numbers and percentages in parenthesis. *p* values were obtained using ^1^ chi-2 tests.

**Table 4:** Pfirrmann grade changes from before to after the intervention.

Variable	Supplement (23 Patients 115 IVDs)	Placebo (22 Patients 110 IVDs)
Improvements regarding Pfirrmann grades (n, %)	9 (39.1)	10 (45.5)
three-grade improvement	1 (4.3)	0
two-grade improvement	3 (13.0)	3 (13.6)
one-grade improvement	5 (21.7)	7 (31.8)
No changes regarding Pfirrmann grades	14 (60.9)	10 (45.5)
Worsening regarding Pfirrmann grades	0	2 (9.1)

Abbreviations: IVDs, intervertebral discs.

**Table 5:** Mean volume (in mm^3^) before and after the 3-month intervention.

Variable	Supplement (23 Patients 115 IVDs)	Placebo (22 Patients 110 IVDs)	*p* Value (between Groups)
Mean volume (in mm^3^)			
before intervention after intervention	18,621.1 ± 4181.4 19,361.4 ± 4489.3	17,805.5 ± 4547.9 17,388.3 ± 4369.7	0.534 ^1^ 0.143 ^1^
*p*-value (within groups)	<0.001 ^2^	0.036 ^2^	-

Abbreviations: IVDs, intervertebral discs. Values of continuous variables are presented as means ± standard deviation. *p* values were obtained using ^1^ independent *t*-test and ^2^ paired *t*-tests.

## Data Availability

The datasets used and analyzed in this study are available from the corresponding author on reasonable request.

## Supplementary Materials

The following supporting information can be downloaded at: https://www.mdpi.com/article/10.3390/nu16162695/s1, Table S1. CONSORT checklist; Table S2. Participant inclusion and exclusion criteria; Table S3: IVD degeneration according to Pfirrmann; Table S4. Cohen’s Kappa and its 95% confidence intervals; Table S5. Distribution of Pfirrmann grades of each intervertebral disc distance; Table S6. Volume (in mm3) of each intervertebral disc distance.

## References

[1] Goggs R., Vaughan-Thomas A., Clegg P.D., Carter S.D., Innes J.F., Mobasheri A., Shakibaei M., Schwab W., Bondy C.A. (2005). Nutraceutical Therapies for Degenerative Joint Diseases: A Critical Review. Crit. Rev. Food Sci. Nutr..

[2] Whatley B.R., Wen X. (2012). Intervertebral disc (IVD): Structure, degeneration, repair and regeneration. Mater. Sci. Eng. C.

[3] Dionne C.E., Laurin D., Desrosiers T., Abdous B., Le Sage N., Frenette J., Mondor M., Pelletier S. (2016). Serum vitamin C and spinal pain: A nationwide study. Pain.

[4] Bruyere O., Zegels B., Leonori L., Rabenda V., Janssen A., Bourges C., Reginster J.Y. (2012). Effect of collagen hydrolysate in articular pain: A 6-month randomized, double-blind, placebo controlled study. Complement. Ther. Med..

[5] Benito-Ruiz P., Camacho-Zambrano M.M., Carrillo-Arcentales J.N., Mestanza-Peralta M.A., Vallejo-Flores C.A., Vargas-Lopez S.V., Villacis-Tamayo R.A., Zurita-Gavilanes L.A. (2009). A randomized controlled trial on the efficacy and safety of a food ingredient, collagen hydrolysate, for improving joint comfort. Int. J. Food Sci. Nutr..

[6] Kalman D.S., Heimer M., Valdeon A., Schwartz H., Sheldon E. (2008). Effect of a natural extract of chicken combs with a high content of hyaluronic acid (Hyal-Joint) on pain relief and quality of life in subjects with knee osteoarthritis: A pilot randomized double-blind placebo-controlled trial. Nutr. J..

[7] Clegg D.O., Reda D.J., Harris C.L., Klein M.A., O’Dell J.R., Hooper M.M., Bradley J.D., Bingham C.O., Weisman M.H., Jackson C.G. (2006). Glucosamine, chondroitin sulfate, and the two in combination for painful knee osteoarthritis. N. Engl. J. Med..

[8] Oesser S., Seifert J. (2003). Stimulation of type II collagen biosynthesis and secretion in bovine chondrocytes cultured with degraded collagen. Cell Tissue Res..

[9] Oesser S., Adam M., Babel W., Seifert J. (1999). Oral administration of (14)C labeled gelatin hydrolysate leads to an accumulation of radioactivity in cartilage of mice (C57/BL). J. Nutr..

[10] Liu D., Nikoo M., Boran G., Zhou P., Regenstein J.M. (2015). Collagen and gelatin. Annu. Rev. Food Sci. Technol..

[11] Balogh L., Polyak A., Mathe D., Kiraly R., Thuroczy J., Terez M., Janoki G., Ting Y., Bucci L.R., Schauss A.G. (2008). Absorption, uptake and tissue affinity of high-molecular-weight hyaluronan after oral administration in rats and dogs. J. Agric. Food Chem..

[12] Persiani S., Rotini R., Trisolino G., Rovati L.C., Locatelli M., Paganini D., Antonioli D., Roda A. (2007). Synovial and plasma glucosamine concentrations in osteoarthritic patients following oral crystalline glucosamine sulphate at therapeutic dose. Osteoarthr. Cartil..

[13] Persiani S., Roda E., Rovati L.C., Locatelli M., Giacovelli G., Roda A. (2005). Glucosamine oral bioavailability and plasma pharmacokinetics after increasing doses of crystalline glucosamine sulfate in man. Osteoarthr. Cartil..

[14] Henrotin Y., Mobasheri A., Marty M. (2012). Is there any scientific evidence for the use of glucosamine in the management of human osteoarthritis?. Arthritis Res. Ther..

[15] Akeda K., Yamada T., Inoue N., Nishimura A., Sudo A. (2015). Risk factors for lumbar intervertebral disc height narrowing: A population-based longitudinal study in the elderly. BMC Musculoskelet. Disord..

[16] Higa J.K., Panee J. (2011). Bamboo extract reduces interleukin 6 (IL-6) overproduction under lipotoxic conditions through inhibiting the activation of NF-kappaB and AP-1 pathways. Cytokine.

[17] Schulz K.F., Altman D.G., Moher D., Group C. (2011). CONSORT 2010 statement: Updated guidelines for reporting parallel group randomised trials. Int. J. Surg..

[18] Frestedt J.L., Walsh M., Kuskowski M.A., Zenk J.L. (2008). A natural mineral supplement provides relief from knee osteoarthritis symptoms: A randomized controlled pilot trial. Nutr. J..

[19] Giordano N., Fioravanti A., Papakostas P., Montella A., Giorgi G., Nuti R. (2009). The efficacy and tolerability of glucosamine sulfate in the treatment of knee osteoarthritis: A randomized, double-blind, placebo-controlled trial. Curr. Ther. Res. Clin. Exp..

[20] Usha P.R., Naidu M.U. (2004). Randomised, Double-Blind, Parallel, Placebo-Controlled Study of Oral Glucosamine, Methylsulfonylmethane and their Combination in Osteoarthritis. Clin. Drug Investig..

[21] Mannion A.F., Junge A., Fairbank J.C., Dvorak J., Grob D. (2006). Development of a German version of the Oswestry Disability Index. Part 1: Cross-cultural adaptation, reliability, and validity. Eur. Spine J..

[22] Mannion A.F., Junge A., Grob D., Dvorak J., Fairbank J.C. (2006). Development of a German version of the Oswestry Disability Index. Part 2: Sensitivity to change after spinal surgery. Eur. Spine J..

[23] Ware J., Kosinski M., Keller S.D. (1996). A 12-Item Short-Form Health Survey: Construction of scales and preliminary tests of reliability and validity. Med. Care.

[24] Bull F.C., Maslin T.S., Armstrong T. (2009). Global physical activity questionnaire (GPAQ): Nine country reliability and validity study. J. Phys. Act. Health.

[25] Pfirrmann C.W., Metzdorf A., Zanetti M., Hodler J., Boos N. (2001). Magnetic resonance classification of lumbar intervertebral disc degeneration. Spine.

[26] Sharma A., Lancaster S., Bagade S., Hildebolt C. (2014). Early pattern of degenerative changes in individual components of intervertebral discs in stressed and nonstressed segments of lumbar spine: An in vivo magnetic resonance imaging study. Spine.

[27] Huemer D. (2024). Magnetresonanztomographie-Vermessene Veränderungen der Disci Intervertebrales nach 3-Monatiger Nahrungsergänzungsmitteleinnahme von Patienten mit Lumbaler Osteochondrose. Master’s Thesis.

[28] Kim C.W., Doerr T.M., Luna I.Y., Joshua G., Shen S.R., Fu X., Wu A.M. (2016). Minimally Invasive Transforaminal Lumbar Interbody Fusion Using Expandable Technology: A Clinical and Radiographic Analysis of 50 Patients. World Neurosurg..

[29] Qaseem A., Wilt T.J., McLean R.M., Forciea M.A., Denberg T.D., Barry M.J., Boyd C., Chow R.D., Fitterman N., Clinical Guidelines Committee of the American College of Physicians (2017). Noninvasive Treatments for Acute, Subacute, and Chronic Low Back Pain: A Clinical Practice Guideline from the American College of Physicians. Ann. Intern. Med..

[30] Reme S.E., Tveito T.H., Harris A., Lie S.A., Grasdal A., Indahl A., Brox J.I., Tangen T., Hagen E.M., Gismervik S. (2016). Cognitive Interventions and Nutritional Supplements (The CINS Trial): A Randomized Controlled, Multicenter Trial Comparing a Brief Intervention with Additional Cognitive Behavioral Therapy, Seal Oil, and Soy Oil for Sick-Listed Low Back Pain Patients. Spine.

[31] Gagnier J.J. (2008). Evidence-informed management of chronic low back pain with herbal, vitamin, mineral, and homeopathic supplements. Spine J..

[32] Zadro J.R., Shirley D., Ferreira M., Carvalho Silva A.P., Lamb S.E., Cooper C., Ferreira P.H. (2018). Is Vitamin D Supplementation Effective for Low Back Pain? A Systematic Review and Meta-Analysis. Pain Physician.

[33] Jensen O.K., Andersen M.H., Ostgard R.D., Andersen N.T., Rolving N. (2019). Probiotics for chronic low back pain with type 1 Modic changes: A randomized double-blind, placebo-controlled trial with 1-year follow-up using Lactobacillus Rhamnosis GG. Eur. Spine J..

[34] Sha S., Chen L.J., Brenner H., Schottker B. (2024). Serum 25-Hydroxyvitamin D Status and Vitamin D Supplements Use Are Not Associated with Low Back Pain in the Large UK Biobank Cohort. Nutrients.

[35] Ko S., Kim H.C., Kwon J. (2023). The effectiveness of vitamin D3 supplementation in improving functional outcome of non-surgically treated symptomatic lumbar spinal stenosis: Randomized controlled clinical trial—Pilot study. Medicine.

[36] Hayden J.A., Ellis J., Ogilvie R., Malmivaara A., van Tulder M.W. (2021). Exercise therapy for chronic low back pain. Cochrane Database Syst. Rev..

[37] Yuan Q.L., Guo T.M., Liu L., Sun F., Zhang Y.G. (2015). Traditional Chinese medicine for neck pain and low back pain: A systematic review and meta-analysis. PLoS ONE.

[38] Coulter I.D., Crawford C., Hurwitz E.L., Vernon H., Khorsan R., Suttorp Booth M., Herman P.M. (2018). Manipulation and mobilization for treating chronic low back pain: A systematic review and meta-analysis. Spine J..

[39] Machado G.C., Maher C.G., Ferreira P.H., Day R.O., Pinheiro M.B., Ferreira M.L. (2017). Non-steroidal anti-inflammatory drugs for spinal pain: A systematic review and meta-analysis. Ann. Rheum. Dis..

[40] van Middelkoop M., Rubinstein S.M., Kuijpers T., Verhagen A.P., Ostelo R., Koes B.W., van Tulder M.W. (2011). A systematic review on the effectiveness of physical and rehabilitation interventions for chronic non-specific low back pain. Eur. Spine J..

[41] Saragiotto B.T., Maher C.G., Yamato T.P., Costa L.O., Menezes Costa L.C., Ostelo R.W., Macedo L.G. (2016). Motor control exercise for chronic non-specific low-back pain. Cochrane Database Syst. Rev..

[42] Klinger R., Stuhlreyer J., Schwartz M., Schmitz J., Colloca L. (2018). Clinical Use of Placebo Effects in Patients with Pain Disorders. Int. Rev. Neurobiol..

[43] Videman T., Battie M.C., Ripatti S., Gill K., Manninen H., Kaprio J. (2006). Determinants of the progression in lumbar degeneration: A 5-year follow-up study of adult male monozygotic twins. Spine.

[44] Borthakur A., Maurer P.M., Fenty M., Wang C., Berger R., Yoder J., Balderston R.A., Elliott D.M. (2011). T1rho magnetic resonance imaging and discography pressure as novel biomarkers for disc degeneration and low back pain. Spine.

