# Supplementary material for: A Dietary Supplement in the Management of Patients with Lumbar Osteochondrosis: A Randomized, Double-Blinded, Placebo-Controlled Study
Source: Nutrients. 2024 Aug 14;16(16):2695. doi: 10.3390/nu16162695 (PMC11357631; doi:10.3390/nu16162695)
Supplement: Supplementary file 1 [file nutrients-16-02695-s001.zip › nutrients-3131170-supplementary.pdf]

SUPPLEMENTARY INFORMATION

Article

A Dietary Supplement In The Management Of Patients With Lumbar Osteochondrosis: A Randomized, Double-Blinded, Placebo-Controlled Study

Brenda Laky [1,2,3,4,5*], Daniel Huemer [1,6], Martin Eigenschink [1,7], Benedikt Sagl [4], Rainer Thell [6,8], Karl-Heinz Wagner [5], Werner Anderl [1,9], and Philipp R. Heuberer [1,10]

1 Austrian Research group for Regenerative and Orthopedic Medicine (AURROM), Vienna, Austria; brenda.laky@aurrom.org (BL); danielhuemer.medical@gmail.com (DH); martin.eigenschink@gmail.com (ME); philipp@heuberer.at (PRH); werner@anderl.at (WA)
2 Austrian Society of Regenerative Medicine (RegMed), Vienna, Austria; brenda.laky@regmedaustria.org
3 Faculty of Medicine, Sigmund Freud Private University Medicine, Vienna, Austria; brenda.laky@med.sfu.ac.at
4 Center for Clinical Research, University Clinic of Dentistry, Medical University of Vienna, Austria; brenda.laky@meduniwien.ac.at (BL); benedikt.sagl@meduniwien.ac.at (BS)
5 Department of Nutritional Sciences, the University of Vienna, Austria; karl-heinz.wagner@univie.ac.at
6 Medical University of Vienna, Austria; danielhuemer.medical@gmail.com; rainer.thell@meduniwien.ac.at
7 Department for Trauma and Orthopedic Surgery, AUVA Trauma Center Vienna-Meidling, Vienna, Austria; martin.eigenschink@gmail.com
8 Emergency Department, Klinik Donaustadt, Langobardenstraße 122, 1220 Vienna, Austria; rainer.thell@gesundheitsverbund.at
9 Momentum, Mödling, Austria; werner@anderl.at
10 OrthoCare and HealthPi Medical Center, Vienna, Austria; philipp@heuberer.at

* Correspondence: brenda.laky@aurrom.org ; ORCID: 0000-0003-1198-4132; AURROM: Hartmanngasse 15/9, 1050, Vienna, Austria

Abstract: Various nutritional supplements are available over the counter, yet few have been investigated in randomized controlled trials. The rationale for using the specific mix of nutritional substances including collagen type II, hyaluronic acid, n-acetyl-glucosamine, bamboo extract, L-lysine, and vitamin C is the assumption that combining naturally occurring ingredients of intervertebral disc would maintain spine function. The aim of this double-blind, placebo-controlled randomized trial was to evaluate the efficacy of the nutraceutical supplement-mix in the management of lumbar osteochondrosis. Fifty patients were randomly assigned to either the supplement or placebo group in a 1:1 ratio. Patient-Reported Outcome Measures (PROMs) included the Oswestry Disability Index (ODI), visual analogue scale for pain (pVAS); short form-12 (SF-12) physical and mental component summery subscale scores (PCS and MCS, respectively), and global physical activity questionnaire (GPAQ). Magnetic resonance images (MRI) were used to evaluate degenerative changes of intervertebral discs (IVD) including Pfirrmann grades as well as 3-dimensional (3D) volume measurements. Data were collection at baseline and after the 3-month intervention. None of the PROMs were significantly different between the supplement and placebo group. Disc degeneration according to Pfirrmann classifications remained stable during the 3-month intervention in both groups. Despite no significance regarding the distribution of Pfirrmann grade changes (improvement, no change, worsening; $p=0.259$), in the supplement group, one patient achieved a 3-grade improvement and worsening of Pfirrmann grades were only detected in the placebo group (9.1%). Furthermore, in depth evaluations of MRIs showed significant higher 3D-measured volume changes (increase) in the supplement (+740,3±796,1 mm^3) compared to lower 3D-measured volume changes (decrease) in the placebo group (-417,2±875,0 mm^3; $p<0.001$). In conclusion, this multi-nutrient supplement might not only stabilize progression of lumbar osteochondrosis, as present data demonstrated, it might also potentially even increase IVD-volumes as detected on MRIs.

Table S1. CONSORT checklist

		Reporting Item	Page Number
**Title and Abstract**			
Title	#1a	Identification as a randomized trial in the title.	1
Abstract	#1b	Structured summary of trial design, methods, results, and conclusions	1
**Introduction**			
Background and objectives	#2a	Scientific background and explanation of rationale	2-4
Background and objectives	#2b	Specific objectives or hypothesis	4
**Methods**			
Trial design	#3a	Description of trial design (such as parallel, factorial) including allocation ratio.	4-5
Trial design	#3b	Important changes to methods after trial commencement (such as eligibility criteria), with reasons	5
Participants	#4a	Eligibility criteria for participants	5
Participants	#4b	Settings and locations where the data were collected	5
Interventions	#5	The experimental and control interventions for each group with sufficient details to allow replication, including how and when they were actually administered	5
Outcomes	#6a	Completely defined prespecified primary and secondary outcome measures, including how and when they were assessed	5-6
Sample size	#7a	How sample size was determined.	7
Sample size	#7b	When applicable, explanation of any interim analyses and stopping guidelines	7
Randomization - Sequence generation	#8a	Method used to generate the random allocation sequence.	5
Randomization - Sequence generation	#8b	Type of randomization; details of any restriction (such as blocking and block size)	5
Randomization - Allocation concealment mechanism	#9	Mechanism used to implement the random allocation sequence (such as sequentially numbered containers), describing any steps taken to conceal the sequence until interventions were assigned	5
Randomization - Implementation	#10	Who generated the allocation sequence, who enrolled participants, and who assigned participants to interventions	5
Blinding	#11a	If done, who was blinded after assignment to interventions (for example, participants, care providers, those assessing outcomes) and how.	5
Blinding	#11b	If relevant, description of the similarity of interventions	5
Statistical methods	#12a	Statistical methods used to compare groups for primary and secondary outcomes	7

Statistical methods	#12b	Methods for additional analyses, such as subgroup analyses and adjusted analyses	7
Outcomes	#6b	Any changes to trial outcomes after the trial commenced, with reasons	7
**Results**			
Participant flow diagram (strongly recommended)	#13a	For each group, the numbers of participants who were randomly assigned, received intended treatment, and were analysed for the primary outcome	8
Participant flow	#13b	For each group, losses and exclusions after randomization, together with reason	8
Recruitment	#14a	Dates defining the periods of recruitment and follow-up	8
Recruitment	#14b	Why the trial ended or was stopped	8
Baseline data	#15	A table showing baseline demographic and clinical characteristics for each group	9
Numbers analysed	#16	For each group, number of participants (denominator) included in each analysis and whether the analysis was by original assigned groups	8
Outcomes and estimation	#17a	For each primary and secondary outcome, results for each group, and the estimated effect size and its precision (such as 95% confidence interval)	9-13
Outcomes and estimation	#17b	For binary outcomes, presentation of both absolute and relative effect sizes is recommended	9-13
Ancillary analyses	#18	Results of any other analyses performed, including subgroup analyses and adjusted analyses, distinguishing pre-specified from exploratory	9-13
Harms	#19	All important harms or unintended effects in each group (For specific guidance see CONSORT for harms)	8
**Discussion**			
Limitations	#20	Trial limitations, addressing sources of potential bias, imprecision, and, if relevant, multiplicity of analyses	15
Interpretation	#22	Interpretation consistent with results, balancing benefits and harms, and considering other relevant evidence	14-15
Registration	#23	Registration number and name of trial registry	4
Generalisability	#21	Generalisability (external validity, applicability) of the trial findings	14-15
**Other information**			
Interpretation	#22	Interpretation consistent with results, balancing benefits and harms, and considering other relevant evidence	14-15
Registration	#23	Registration number and name of trial registry	4
Protocol	#24	Where the full trial protocol can be accessed, if available	n/a
Funding	#25	Sources of funding and other support (such as supply of drugs), role of funders	16

The CONSORT checklist is distributed under the terms of the Creative Commons Attribution License CC-BY. This checklist was completed on 08. July 2024 using https://www.goodreports.org/, a tool made by the EQUATOR Network in collaboration with Penelope.ai

Table S2. Participant inclusion and exclusion criteria

Inclusion criteria
• female and male patients aged between 18 and 75 years of age
• patients with symptomatic (VAS ≥4) MRI-confirmed (Pfirrmann grades 2, 3, and 4; see Table S2) lumbar osteochondrosis
• Given written informed consent form for participation in the study
Exclusion criteria:
• Patients with asymptomatic (VAS ≤ 3) MRI-confirmed (Pfirrmann grades 1 and 5 (see Table S2) lumbar osteochondrosis
• Pathologies
o rheumatoid osteoarthritis
o fibromyalgia
o scoliosis
o neurological deficit
o morbid adiposity (body mass index, BMI ≥40)
o any other current or past clinically significant disease (comorbidity) that, in the opinion of the orthopedic consultant, might confound the results of the study or poses an additional risk to the subject during participation in the study (e.g. renal, neoplastic, epigastric disease,…)
• Previous surgeries of IVD(s), hip(s)
• Allergies/intolerances against any component of the dietary supplement (e.g. shellfishes)
• Pregnant or lactating women
• Not fluent (read/understand) in German (Consent, questionnaires, and study forms are in German)
• Not able to undergo MRI (e.g. claustrophobia, pacemaker,…)

Table S3: IVD degeneration according to Pfirrmann (16)

Grade	Structure	Distinction of nucleus and annulus	Signal intensity	Intervertebral disc height	MRI (from (17))
1	Homogeneous, bright white	Clear	Hyperintense, isointense to CSF	Normal	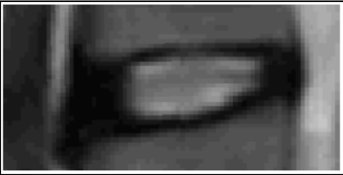
2	Inhomogeneous with or without horizontal bands	Clear	Hyperintense, isointense to CSF	Normal	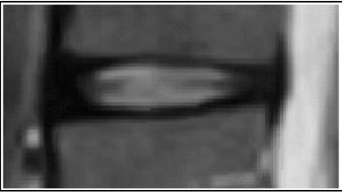
3	Inhomogeneous, gray	Unclear	Intermediate	Normal to slightly decreased	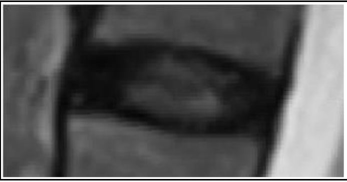
4	Inhomogeneous, gray to black	Lost	Intermediate to hypointense	Normal to moderately decreased	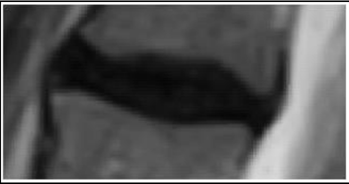
5	Inhomogeneous, black	Lost	Hypointense	Collapsed disc space	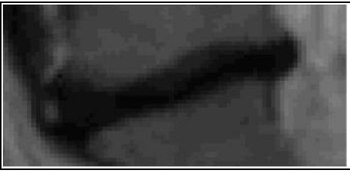

Table S4. Cohen's Kappa and its 95% confidence intervals

	Kappa	Lower 95%CI	Upper 95%CI	p-values
T0 L12	0.838	0.706	0.969	<0.001
T0 L23	0.752	0.595	0.909	<0.001
T0 L34	0.809	0.668	0.951	<0.001
T0 L45	0.781	0.632	0.930	<0.001
T0 L5S1	0.772	0.629	0.915	<0.001
	Kappa	Lower 95%CI	Upper 95%CI	p-values
T1 L12	0.865	0.741	0.990	<0.001
T1 L23	0.749	0.593	0.906	<0.001
T1 L34	0.786	0.642	0.930	<0.001
T1 L45	0.851	0.728	0.975	<0.001
T1 L5S1	0.944	0.868	1.020	<0.001

Abbreviation: CI, confidence interval.

Table S5. Distribution of Pfirrmann grades of each intervertebral disc distance

	MRI before intervention			MRI after intervention		
Nr. of intervertebral discs	**TOTAL (n=225)**	**Supplement (n=115)**	**Placebo (n=110)**	**TOTAL (n=225)**	**Supplement (n=115)**	**Placebo (n=110)**
L1/L2	45	23	22	45	23	22
Pfirrmann grade I	14	6	8	19	8	11
Pfirrmann grade II	21	13	8	17	12	5
**No disc degeneration (Pfirrmann grade I+II)**	**35**	**19**	**16**	**36**	**20**	**16**
Pfirrmann grade III	6	4	2	6	3	3
Pfirrmann grade IV	4	0	4	3	0	3
Pfirrmann grade V	0	0	0	0	0	0
**Disc degeneration (Pfirrmann grade III+IV+V)**	**10**	**4**	**6**	**9**	**3**	**6**
L2/L3	45	23	22	45	23	22
Pfirrmann grade I	8	3	5	9	3	6
Pfirrmann grade II	19	12	7	23	14	9
**No disc degeneration (Pfirrmann grade I+II)**	**27**	**15**	**12**	**32**	**17**	**15**
Pfirrmann grade III	12	7	5	6	4	2
Pfirrmann grade IV	5	0	5	6	1	5
Pfirrmann grade V	1	1	0	1	1	0
**Disc degeneration (Pfirrmann grade III+IV+V)**	**18**	**8**	**10**	**13**	**6**	**7**
L3/L4	45	23	22	45	23	22
Pfirrmann grade I	8	4	4	9	6	3
Pfirrmann grade II	12	6	6	15	6	9
**No disc degeneration (Pfirrmann grade I+II)**	**20**	**10**	**10**	**24**	**12**	**12**
Pfirrmann grade III	20	11	9	16	9	7
Pfirrmann grade IV	4	2	2	4	2	2
Pfirrmann grade V	1	0	1	1	0	1
**Disc degeneration (Pfirrmann grade III+IV+V)**	**25**	**13**	**12**	**21**	**11**	**10**
L4/L5	45	23	22	45	23	22
Pfirrmann grade I	6	4	2	6	4	2
Pfirrmann grade II	6	2	4	7	3	4
**No disc degeneration (Pfirrmann grade I+II)**	**12**	**6**	**6**	**13**	**7**	**6**
Pfirrmann grade III	19	11	8	19	11	8
Pfirrmann grade IV	8	4	4	8	3	5
Pfirrmann grade V	6	2	4	5	2	3
**Disc degeneration (Pfirrmann grade III+IV+V)**	**33**	**17**	**16**	**32**	**16**	**16**
L5/S1	45	23	22	45	23	22
Pfirrmann grade I	6	1	5	7	2	5
Pfirrmann grade II	5	3	2	8	4	4
**No disc degeneration (Pfirrmann grade I+II)**	**11**	**4**	**7**	**15**	**6**	**9**
Pfirrmann grade III	12	5	7	8	4	4
Pfirrmann grade IV	11	8	3	11	7	4
Pfirrmann grade V	11	6	5	11	6	5
**Disc degeneration (Pfirrmann grade III+IV+V)**	**34**	**19**	**15**	**30**	**17**	**13**
**No disc degeneration (Pfirrmann grade I+II)**	**105**	**54**	**51**	**120**	**62**	**58**
**TOTAL Disc degeneration (Pfirrmann grade III+IV+V)**	**120**	**61**	**59**	**105**	**53**	**52**

Table S6. Volume (in mm³) of each intervertebral disc distance

Volume (in mm³)	Supplement (23 patients 115 IVDs)	Placebo (22 patients 110 IVDs)	*P* value[1] (between groups)
Before intervention			
L1-L2	16487.3±4717.2	15657.4±3791.8	0.520
L2-L3	19723.3±5283.6	18298.4±5334.4	0.373
L3-L4	21691.1±5040.8	19520.3±5457.6	0.173
L4-L5	19817.3±5070.4	19002.7±5207.0	0.598
L5-S1	15386.5±4538.9	16548.5±5470.1	0.441
After intervention			
L1-L2	17172.5±4896.0	15251.2±3629.7	0.144
L2-L3	20492.9±5320.4	17802.9±5094.2	0.091
L3-L4	22642.9±5152.1	19483.0±5505.5	0.053
L4-L5	20515.5±5204.4	18475.0±4767.3	0.178
L5-S1	15983.3±5007.0	15929.3±5228.6	0.972
***P* value** (within groups)			
L1-L2	<0.001	0.042	-
L2-L3	0.005	0.051	
L3-L4	<0.001	0.881	
L4-L5	0.019	0.027	
L5-S1	0.062	0.022	

Abbreviations: IVDs, intervertebral discs.
Values of continuous variables are presented as means ± standard deviation.
P values were obtained using [1] independent t-tests and [2] paired t-tests.
